# Positive exercise test?

**DOI:** 10.1007/s12471-016-0863-1

**Published:** 2016-07-12

**Authors:** W. K. den Dekker, J. W. Deckers, S. C. Yap

**Affiliations:** Department of cardiology, Thoraxcenter, ErasmusMedical Center, Rotterdam, The Netherlands

A 46-year-old male was referred for exercise testing after he was evaluated in the emergency department because of progressive fatigue, dyspnoea on exertion and chest pain with tingling of the left arm. He was an otherwise healthy man who was a professional climber. He had no cardiovascular risk factors and was not using any medication. On physical examination he was normotensive and the cardiovascular examination was normal. His troponin level was normal. A bicycle exercise test was performed to rule out ischaemia. He exercised for 13 min and 40 sec, reaching 242 Watt (reference value 208 Watt) with a maximum heart rate of 167 beats/min (96 % of predicted maximal heart rate) and a blood pressure of 187/92 mmHg. The test was stopped because of fatigue. He had no chest pain. Two recordings of the exercise test are shown here (Fig. 1a, b).

## Questions

Is this a positive test for ischaemia?

What is the prognosis?

## Answer

You will find the answer elsewhere in this issue.

**Fig. 1:**
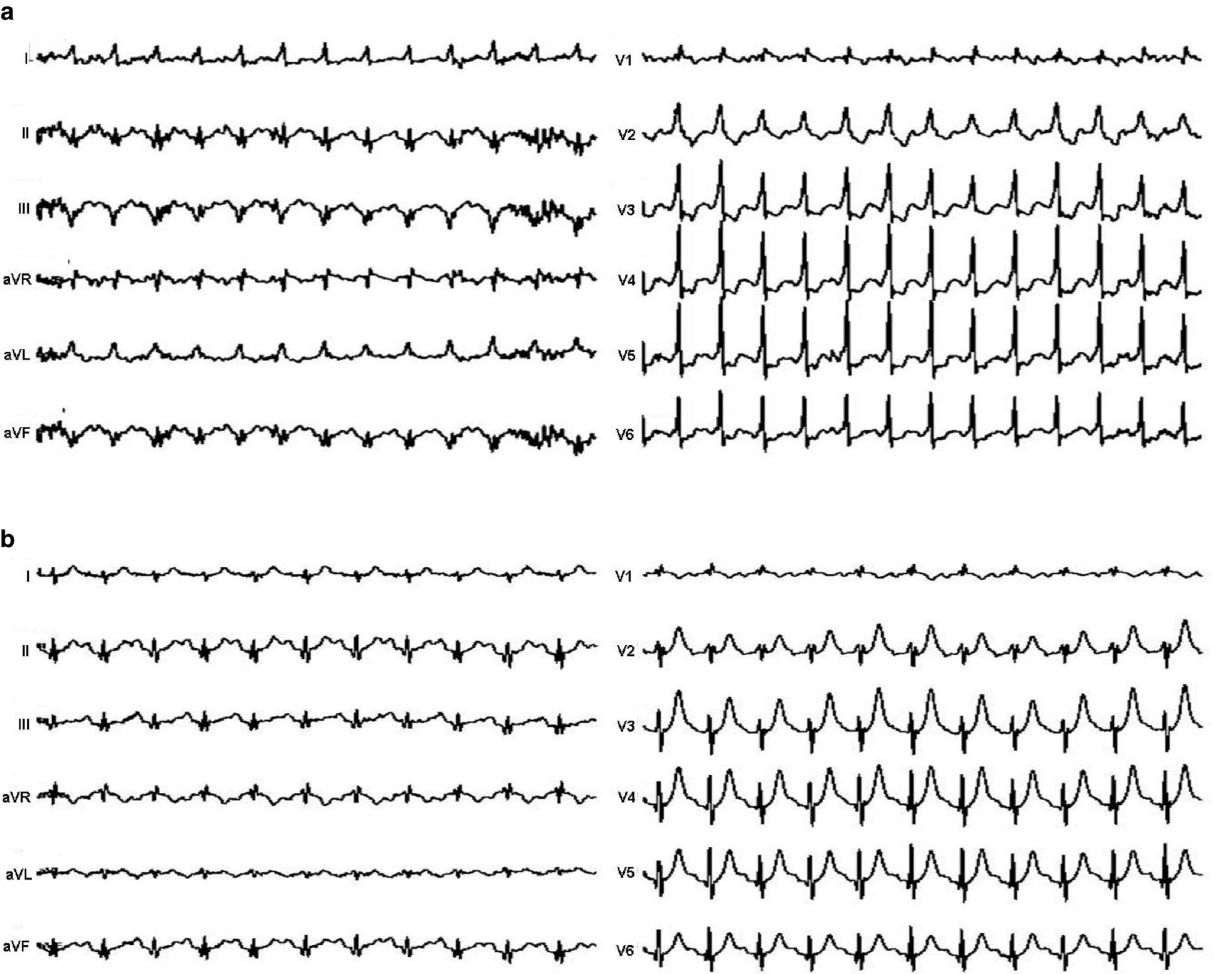
**a** During exercise, **b** Recovery phase

